# Transcriptomics analysis of *in vivo Opisthorchis viverrini*: Stage-specific gene expression and novel genes with stable expression in mammalian stages for opisthorchiasis intervention

**DOI:** 10.1371/journal.pntd.0013714

**Published:** 2025-12-18

**Authors:** Waraporn Taweesin, Siriyakorn Kulwong, Wassana Jamnongkan, Wichit Taron, Autchasai Siriprayong, Sirinya Sitthirak, Anchalee Techasen, Opal Pitaksakulrat, Nisana Namwat, Poramate Klanrit, Watcharin Loilome, Arporn Wangwiwatsin

**Affiliations:** 1 Department of Biochemistry, Faculty of Medicine, Khon Kaen University, Khon Kaen, Thailand; 2 Department of Systems Biosciences and Computational Medicine, Faculty of Medicine, Khon Kaen University, Khon Kaen, Thailand; 3 Cholangiocarcinoma Research Institute, Khon Kaen University, Khon Kaen, Thailand; 4 Department of Medical Technology, Faculty of Associated Medical Sciences, Khon Kaen University, Khon Kaen, Thailand; 5 School of Allied Health Sciences, Walailak University, Nakhon Si Thammarat, Thailand; 6 Department of Parasitology, Faculty of Medicine, Khon Kaen University, Khon Kaen, Thailand; Rio de Janeiro State University: Universidade do Estado do Rio de Janeiro, BRAZIL

## Abstract

*Opisthorchis viverrini*, Southeast Asian liver fluke, is a parasitic flatworm that has been widely spread in Asia and is a major risk factor for bile duct cancer - cholangiocarcinoma (CCA). Over 12 million people are at risk of opisthorchiasis, and the associated CCA is known to have claimed around 20,000 deaths per year in Thailand alone, with the number projected to be underestimated elsewhere. There is an opportunity to develop more efficient drug targets and diagnostic biomarkers, and these are urgently needed. Despite previous studies on gene expression analysis and reference genome, the knowledge on the biological processes of *O. viverrini* during its development remains largely unclear. Our study employed RNA-sequencing transcriptomics of *O. viverrini* developmental stages within hamsters from the juvenile stage (14-day post-infection), the adult stage of acute infection (42 days), and the adult stage of chronic infection (180 days). Differential gene expression and functional analyses were performed, and genes with stable expression (GSEs) were identified based on the transcript per million (TPM) normalization method and coefficient of variation. Our results show that key genes in juveniles were mostly associated with proteolysis, energy metabolism, signal transduction, and development. Significantly up-regulated genes in adult *O. viverrini* were associated with parasitic reproductive systems and parasitism. A total of 2,011 GSEs were identified, with 27 genes being highly expressed, and 628 genes showing no orthologues in the human reference genome. From this novel dataset, we illustrated insight into parasite biology, which revealed key molecular processes during intra-mammalian infection, and provided the candidate targets for biomarkers, drugs, and vaccine development. This valuable information will contribute to opisthorchiasis diagnostics and prevention in endemic countries and provide leads for future characterization of essential genes across liver fluke species.

## Author summary

*Opisthorchis viverrini*, a liver fluke parasite, infects millions of people in Southeast Asia and is a leading cause of bile duct cancer, a disease with high fatality rates. People become infected by eating raw or undercooked freshwater fish carrying the parasite. Chronic infections often go unnoticed but can cause severe liver damage and inflammation, increasing the risk of cancer over time. Current diagnostic methods have improved, but still face challenges, such as false positives caused by similar parasites. Better tools for diagnosing and understanding this parasite are urgently needed to reduce its impact on public health. In this study, we examined how the parasite’s genes are turned on and off during its life stages inside a mammalian host. By analyzing parasites from an established hamster model, we identified genes that are active in the parasite during early infection, adulthood, and long-term chronic infection. We also discovered over 2,000 genes that the parasite used during infection in a mammalian host. These genes include some that are not found in humans and might be promising specific targets for intervention. Our findings provide a roadmap for developing more precise diagnostic tools and potential therapies, helping to combat this neglected tropical disease and its deadly complications.

## Introduction

*Opisthorchis viverrini* is a food-borne parasitic flatworm (liver fluke) that causes an infection known as opisthorchiasis. Opisthorchiasis has been widely spread in countries around the Great Mekong Subregion river, including Thailand, Laos, Cambodia, and Vietnam, and is a major risk factor for bile duct cancer, or cholangiocarcinoma (CCA) [1]. To develop their life cycles, *O. viverrini* typically reside in three major diverse hosts for the different developmental stages, which include snails (the first intermediate hosts for the cercariae stage), fishes (the second intermediate hosts for the metacercarial stage), and humans that acts as the last infective hosts for adult worms. Following the infection to human contracted by consuming raw infective freshwater fish, or salted preserved fish contaminated with *O. viverrini* metacercaria, left untreated, chronic liver fluke infection can eventually lead to CCA development driven by many infection-associated factors including the roles of liver fluke’s excretory/secretory (ES) products, proteases, and inflammation-induced DNA damage. [2,3]. Recent published statistics of opisthorchiasis and CCA prevalence during 2013–2019 showed that over 85,323 (32.37% of total 263,588 participants) people are at risk of opisthorchiasis, and the associated CCA is diagnosed at 948 individuals (0.36% of all participants) [4], and claiming around 39–61 deaths per 100,000 in 2005 in Thailand alone [5], Furthermore, the traditional ingestion of raw freshwater fish infected with *O. viverrini* is a major factor attributing to opisthorchiasis, which account for approximately 12 million people in Thailand, Laos, Cambodia, and Vietnam [6]. This high infection is strongly associated with CCA, causing approximately 20,000 deaths annually [1].

Current interventions involve routine urine- or stool-based diagnosis and treatment with praziquantel (PZQ). Although PZQ treatment is effective, only the adult stage responds effectively to PZQ [7], and re-infection can increase the risk of CCA development [8]. Nowadays, molecular-based detection of *O. viverrini* antigens using urinary samples is more comfortable and more rapid than fecal egg count examinations. What is more, the molecular-based method has demonstrated higher sensitivity, specificity, and consistency for the diagnostic and screening of *O. viverrini* infection [9,10]. However, the latest antigen-based detection still has some cross-species reactions [11]. Therefore, a comprehensive understanding of parasite biology is necessary for developing effective and suitable diagnosis and disease intervention strategies, including new target drugs or vaccine discovery, candidate biomarkers for opisthorchis diagnosis, and prevention of helminth infection.

Omics has been a valuable technique to study various biomolecular processes and comprehend the complex relationships among DNAs, RNAs, proteins, and metabolites by the integration of multiple omics [12]. Particularly, transcriptomics through RNA-seq analysis enables the profiling of all transcripts and their underlying molecular mechanisms, providing valuable insights into how gene expression is modulated in response to pathological conditions within cells or organisms [13–15]. Previous transcriptomic work allows us to understand some molecular mechanisms of *O. viverrini* from juvenile (14 days post-infection) to adult (42 days infection). However, the study’s scope was limited in its exploration of host-parasite interactions, and life stage-specific data, but lacked worms from the chronic infection stage, and no clear description of biological replicates [16]. Since the first genome of *O. viverrini* was successfully characterized and provided the insight into parasite biology as well as the association of CCA and *O. viverrini* [17]*,* the gene information derived by this genome were applied for serving more in-depth research goals; for instance, to understand the effects of gene expression from *O. viverrini* in diabetic hamsters for opisthorchiasis control in diabetic patients [18]. Furthermore, based on this reference genome, several studies were able to investigate molecular mechanisms through specific proteins in *O. viverrini*; for instance, the role of serpins and cystatins in fibrinolysis and cysteine proteinase activity has been explored [19,20]. Nevertheless, the comprehensive molecular mechanism expressed by *O. viverrini*, investigated via the lens of a comprehensive transcriptomics study, remains lacking.

To fill these knowledge gaps, and enlist novel candidate biomarkers as well as potential drug targets, we conducted RNA-seq analysis to identify differentially expressed genes in mammalian stages during a developmental timecourse from the hamster model, encompassing *ex vivo O. viverrini* 14-days, 42-days, and 180-days post-infection. Moreover, for the first time, we have revealed the list of *O. viverrini* housekeeping genes during the mammalian stage as well as proposing stage-specific candidate genes for the further characterization and development of drug and vaccine targets, key biomarkers for opisthorchis prevention and diagnosis.

## Methods

### Ethics statement

All animal procedures were conducted according to protocols approved by the Institutional Animal Care and Use Committee of Khon Kaen University, based on the Ethical Principles and Guidelines For The Procedures on Animals for Scientific Purposes of the National Research Council of Thailand, with the project license number IACUC-KKU-25/65.

### Host infection and *O. viverrini* collection

The collection of *O. viverrini* metacercaria from cyprinoid fish was performed using pepsin digestion [21]. In brief, the fish were finely cut using a household blender machine for 3–5 minutes. The semi-fluid mass was then further digested using a mixture of 0.25% pepsin (Wako Pure Chemical Industries, Osaka, Japan) and 1.50% HCl (RCI Labscan) in 0.85% NaCl solution, incubated at 37–40 °C in a water bath for one and a half hours with regular stirring. After the incubation, the mixture was filtered through 800, 300, 106, and 250 µm wire mesh sieves, respectively. The final pass-through, which contained metacercaria, was left to precipitate for 5–10 minutes in a beaker or until the supernatant became clear, then the supernatant was removed. Half of the beaker containing the metacercaria was filled with 0.85% NaCl solution to wash out the residual pepsin and HCl, and left for 5–10 minutes for the metacercaria to precipitate. Finally, the metacercaria was transferred to a Petri dish under a stereo microscope for further separation of the parasite from other fish debris.

**Fig 1:**
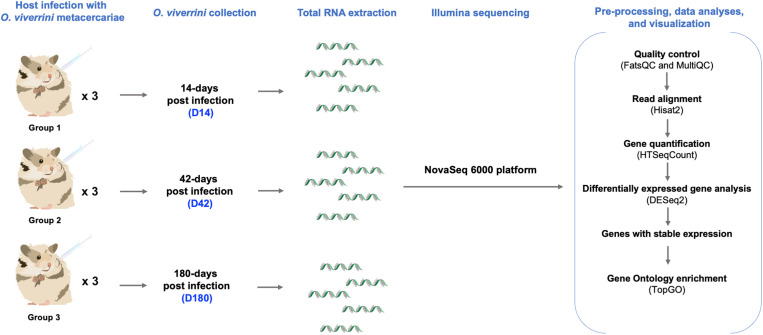
Study design of *O. viverrini* transcriptomics. Golden Syrian hamsters were used as hosts for *O. viverrini* infection. Parasites were collected at three timepoints post-infection for total RNA extraction and mRNA sequencing. RNA-seq data were analyzed using established bioinformatic tools.

The *O. viverrini* metacercaria were used for the infection in hamsters within 24 hours of isolation. Golden Syrian hamsters, MDKKU strain, were divided into three groups according to timepoints of 14 days, 42 days, and 180 days parasite collection post-infection, which reflected the early, acute, and chronic *O. viverrini* infection within the mammalian host. The D14 and D42 conditions might reflect important transcriptome changes in juvenile and acute adult stages [16] while the 6-month samples reflect chronic stages linked to host immune suppression, liver pathogenesis, and ultimately led to CCA development in the mammalian host [22,23]. The individual hamster was infected with 50 metacercaria using 25 ga intragastric tube via oral gavage. The hamsters were maintained at the Animal House Facility, Faculty of Medicine, Khon Kaen University, Thailand. At each timepoint of sample collection, the hamsters were euthanized with an overdose of isoflurane (Sigma Aldrich). *O. viverrini* was recovered from the bile duct of infected hamsters at different timepoints by gently squeezing the liver and observing under a stereo microscope. To preserve the *O. viverrini* for transcriptomes, the worms were washed in normal saline (Sigma Aldrich), and the pool of worms from each hamster, considered one replicate, were stored in 1 ml of TRIzol (Invitrogen) at -80 °C until RNA extraction. Overall workflow of this study is illustrated in Fig 1.

### RNA extraction

Total RNAs of worms from each condition were extracted following the standard TRIzol-chloroform method, followed by RNA Clean & Concentrator-5 kit (Zymo Research). Briefly, the parasite samples stored in -80 °C were thawed on ice; samples were gently pipetted to mix and transferred to a new 2 ml MagNa tube containing ceramic beads (Roche). Homogenization by bead-beating machine was performed at maximum speed for 20 seconds, rested on ice for 1 minute, and then another 20 seconds at maximum speed. Then, the samples were transferred back to a LoBind microfuge tube (Eppendorf). In phase preparation step, 200 μl chloroform was added per 1 mL of TRIzol reagent, and the sample was mixed vigorously by shaking for 5 seconds, incubated at room temperature for 2–3 minutes, and centrifuged at 12,000xg at 4 °C for 15 minutes. To collect the RNA, the upper aqueous phase was transferred by gentle pipetting to a new LoBind microfuge tube, an equal volume of 100% ethanol (RCI Labscan Group) was added, and the mixture was mixed well by pipetting. The mixture was immediately transferred to a Zymo-Spin column, centrifuged and washed according to the Zymo Clean & Concentrator protocol, and eventually eluted with 20 μl DNase/RNase-free water. The extracted RNA was quantified using a NanoDrop spectrophotometer, and its integrity was evaluated using agarose gel electrophoresis and 4150 Agilent TapeStation.

### RNA-sequencing

The extracted total RNAs under all of 3 conditions, including 14-days (D14), 42-days (D42), and 180-days (D180) post-infection, were sent to a Biomarker Technologies (BMKGENE) company in Beijing, China for mRNA sequencing with 150 bp read length, paired-end PE150 using the Illumina NovaSeq 6000 platform to obtain approximately 6 GB of data per sample. Subsequently, the obtained RNA-seq outputs were used for data analyses, and the raw data have been deposited at ENA under accession number ERP150740.

### Pre-processing of RNA-seq data

The RNA-seq data at three timepoints in FASTQ file formats were used to perform quality control (QC) with FastQC [24] and MultiQC tools [25], using Phred score of > 30. For read mapping, the RNA-seq data were aligned to the indexed reference genome of *O. viverrini* obtained from WormBase Parasite, version WBPS19 [17], using Hisat2 (version 2.2.1) [26]. The resulting Sequence Alignment Map (SAM) file was subsequently converted into a Binary Alignment Map (BAM) format using SAMtools (version 1.12) [27]. Gene counts were quantified using HTSeq-count (version 1.99.2) [28] against the reference Genetic Feature Format version 3 (GFF3) annotation of *O. viverrini* obtained from WormBase Parasite version WBPS19 with default parameters, and mapped to coding sequence regions (CDS). Read count tables for all samples (S1 File) were then imported into the R program (version 4.2.3) [29].

### Differential expression analysis

The DESeq2 package (version 1.38.3) [30] was used for pairwise comparisons between D14 versus D42, D14 versus D180, and D42 versus D180. All CDS counts files were used to generate metadata, which included information about sample name, file name, conditions (D14, D42, and D180), and their replicates. The DESeq2 objects were then created through an HTSeq counts matrix, normalized, and transformed into the rlog value using the DESeq2 package. The r-log-transformed values were used to perform multivariate analysis, represented by principal component analysis (PCA). The analysis of DEG was performed with the Wald test statistics with absolute log_2_ fold change (log2FC) greater than 1, and the adjusted p-value less than 0.01 as a cut-off for calling significantly up- and down-regulated genes. DEG results were displayed using ggplot2 package (version 3.4.4) [31] and R program (version 4.2.3).

### Genes with Stable Expression of *O. viverrini* (GSEs)

To identify the GSE of *O. viverrini*, the non-normalized CDS counts stored in DESeq2 objects were derived from every condition and replicates for TPM normalization following the formula (1) [32]. The information on gene length in kilobases (Kb), the gene or exon length of *O. viverrini,* was retrieved from BioMart on WormBase Parasite version WBPS19 [33].


$$\text{Transcript}\;\text{per}\;\text{million}\;\text{or}\;\text{TPM}\;=\;\frac{\text{Non}-\text{normalised}\;\text{CDS}\;\text{count}\;/\;\text{gene}\;\text{length}}{\text{Sum}\;(\text{non}-\text{normalised}\;\text{count}\;/\;\text{gene}\;\text{length})\;}\;\times \;{\text{10}}^{\text{6}}$$ (1)

All TPM-normalized data were used for gene expression variation measured in coefficient of variation (CV), as displayed in formula (2). If a gene has a CV value that is less than or equal to 0.15, the gene is assigned as GSE [34,35].


$$\text{Coefficient}\;\text{of}\;\text{variation}\;\text{or}\;\text{CV}\;=\;\frac{\text{Standard}\;\text{deviation}\;\text{of}\;\text{TPM}}{\text{Mean}\;\text{of}\;\text{TPM}}$$ (2)

### Bioinformatic investigation of *O. viverrini* genes

All interesting genes of *O. viverrini* were searched on databases and publicly available bioinformatic tools to confirm both orthologues and non-orthologues to other species, as well as the similarity of protein sequence. To validate the non-human orthologues in *O. viverrini*, the interesting gene ID list was searched on WormBase Parasite version WBPS19 BioMart [33]. Selected outputs of interest were exported as *O. viverrini* gene ID, gene description, human orthologues ID, human orthologues name, % identity, etc. To confirm orthologues to another species, the gene ID list was used with a similar pipeline with a specific species name. Furthermore, gene sequences were further investigated using Basic Local Alignment Search tool (BLAST) [36]

### Gene Ontology (GO) enrichment analysis

The GO enrichment was performed to assess functional analysis of both DEGs and GSEs using the topGO package (version 2.50.0) [37]. The GO term reference of the *O. viverrini* was acquired via BioMart on WormBase Parasite. The workflow of data pre-processing, DEG analysis, and GO term enrichment analysis followed a pipeline similar to those conducted in previously published studies [38–40].

## Results

### General statistics of RNA-seq data

The RNA-seq was performed on *O. viverrini* samples collected at 14, 42, and 180 days post-infection from experimentally infected Golden Syrian hamsters (Table 1). The sequencing platform generated an average of ~30 gigabase (Gb) for all nine samples, resulting in 19–23 million reads per sample. The RNA-seq quality control results using FASTQC demonstrated the good quality in base calling, with quality scores exceeding 30 in all samples. The GC content was 47%, which is consistent with that of the *O. viverrini* coding sequences previously published [16,17]. Furthermore, approximately ninety percent of the generated RNA-seq reads were successfully mapped to the current reference genome of *O. viverrini* (Table 1), indicating a clean sample collection.

**Table 1:** Sequencing output parameters of *O. viverrini* RNA-seq data.

Condition	The number of worms collected from each hamster	Sample name	% GC content	Total sequenced reads	Percentage of reads mapped to *O. viverrini* reference genome
D14	19	Ov_reads_1_1.sam	47	22,292,275	92.15
D14	17	Ov_reads_1_2.sam	47	21,185,106	91.62
D14	13	Ov_reads_1_3.sam	47	21,878,175	90.02
D42	37	Ov_reads_2_1.sam	47	21,093,539	83.30
D42	31	Ov_reads_2_2.sam	47	21,855,661	91.53
D42	31	Ov_reads_2_3.sam	47	19,168,246	91.19
D180	26	Ov_reads_3_1.sam	47	21,591,435	92.32
D180	27	Ov_reads_3_2.sam	47	21,024,778	92.44
D180	25	Ov_reads_3_3.sam	47	23,377,363	92.38
% Average reads mapped to genome					90.77

### Transcriptomics profiles of *O. viverrini* during intramammalian infection

**Fig 2:**
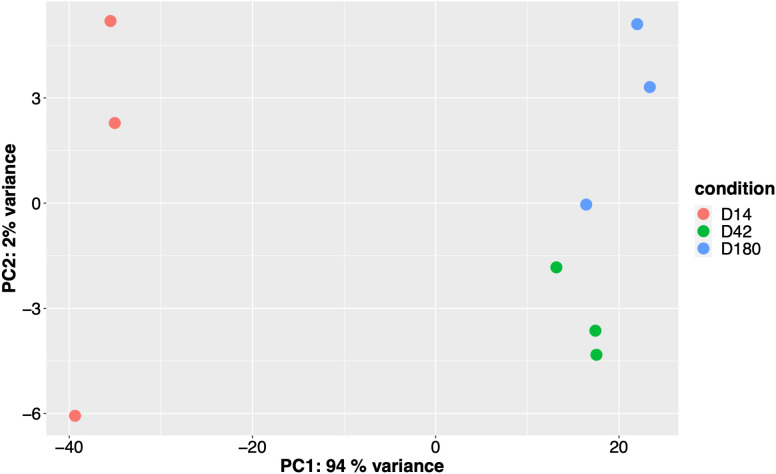
Principal component analysis of the *O. viverrini* RNA-seq data. The PCA plot showed the RNA-seq data clustering based on gene expression profiles using rlog-transformed values derived from DESeq2-normalized counts. Each data point represents a biological replicate, a pool of worms collected from one hamster host, and the color indicated the timepoints of sample collection.

To comprehensively investigate the parasite biology of *O. viverrini* over developmental stages in a mammalian host, we performed principal component analysis (PCA) on the rlog-transformed gene read count using DESeq2 [30]. Consistent with our expectations, the PCA revealed most RNA-seq data captured by the first principal component (PC1), with 94% of total variance in the data on the PC1 axis. Conversely, the second principal component (PC2) explained only 2% of the variance (Fig 2). The PCA analysis indicates that the gene expression profiles were distinctly different between the juvenile (D14) and adults (D42 and D180). The two adult groups exhibited a complementary transcriptome profile demonstrated by their close proximity clustering; yet the replicates within each group suggested that there were some inter-group differences.

**Fig 3:**
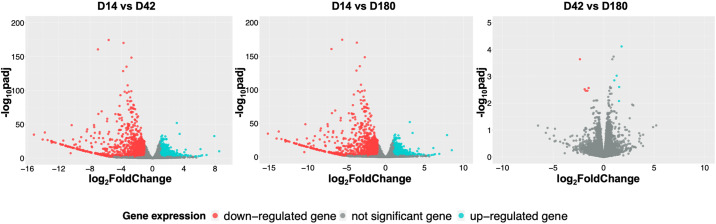
Pairwise comparisons between all timepoints. Volcano plots of pairwise comparisons of differential gene expressions of D14 *vs* D42 (juvenile worms compared to adult worms from acute infection stage), D14 *vs* D180 (juvenile worms compared to adult worms from chronic infection stage), and D42 *vs* D180 (adult worms from acute stage compared to those from chronic infection stage). The light blue dots indicate up-regulated genes with log2FC > 1 and adjusted p-value < 0.01. The red dots were the down-regulated genes with log2FC < -1 and adjusted p-value < 0.01. Genes that failed either log2FC or adjusted p-value cut-offs were indicated in grey. Detailed results of all pairwise comparison is available in S1–S3 Tables.

Numerous differentially expressed genes were identified when gene expression between juvenile and adult stages was compared. At D14, the juvenile worms up-regulated 524 genes in comparison to D42, whereas the D42 worms up-regulated 1,255 genes (Fig 3 and S1 Table). In a pairwise comparison between D14 and D180, 905 and 1,744 up-regulated genes in D14 and D180, respectively (Fig 3 and S2 Table). Finally, between the adult worms from acute and chronic phases of infection, D42 versus D180, only a small number of differentially expressed genes were identified; five genes were up-regulated in D42 worms and another five genes were up-regulated in D180 worms (Fig 3 and S3 Table).

Amongst the differentially expressed genes, there were lots of known genes that may play key roles in the fundamental biological functions of parasitic worms within mammalian hosts. In order to comprehensively describe the infection biology of *O. viverrini*, we investigated at each timepoint the top up-regulated genes that may directly influence their biology.

As displayed in Table 2, multiple genes associated with the biological process of the breakdown of proteins or peptides into smaller amino acids were up-regulated in D14 compared to D42 *O. viverrini*. All these genes represent log2FC between 3.08 and 7.89 (approximately 8–237 fold higher expression in D14), such as *peptidase A1 domain-containing protein* (T265_11050, T265_11760, and T265_13674), *peptidase C13 family protein* (T265_09482, T265_11933), and legumain (T265_10755, T265_12094, T265_15375, and T265_15407)

**Table 2:** Top up-regulated genes in D14 compared to D42 with log2FC > 3.

Gene ID	Log2FC	Adjusted p-value	Gene name
T265_11595	8.50	1.18E-10	Secreted protein
T265_11050	7.89	4.17E-33	Peptidase A1 domain-containing protein^a^
T265_11760	6.89	3.68E-05	Peptidase A1 domain-containing protein^a^
T265_10009	6.33	1.10E-04	C-type lectin domain-containing protein
T265_04509	6.13	7.19E-04	Homeobox domain-containing protein
T265_13674	6.13	7.53E-14	Peptidase A1 domain-containing protein^a^
T265_12117	5.88	2.28E-03	–
T265_05132	5.54	2.74E-03	BLOC-1-related complex subunit 5
T265_14809	5.44	7.17E-03	PI3K/PI4K domain-containing protein
T265_15271	4.88	3.54E-04	Tetraspanin
T265_07894	4.58	1.96E-04	Iron ABC transporter permease
T265_15680	4.46	4.90E-04	SMB domain-containing protein
T265_12094	4.34	1.04E-04	Legumain^a^
T265_10755	4.25	2.53E-05	Legumain^a^
T265_05905	4.23	4.46E-13	SERPIN domain-containing protein
T265_01174	4.15	7.69E-05	Usp domain-containing protein
T265_02179	4.06	3.16E-09	Periplasmic binding protein
T265_05858	3.98	1.39E-03	Cadherin domain protein
T265_11933	3.81	4.43E-05	Peptidase C13 family protein^a^
T265_03732	3.64	3.83E-03	Zinc finger, C2H2 type
T265_15287	3.59	9.90E-03	Ig-like domain-containing protein
T265_02178	3.56	1.27E-05	PLCXc domain-containing protein
T265_08985	3.56	1.38E-06	EF hand
T265_02180	3.49	6.21E-03	Usp domain-containing protein
T265_05750	3.48	1.27E-05	TGF_BETA_2 domain-containing protein
T265_09482	3.46	2.27E-36	Peptidase C13 family protein^a^
T265_03231	3.42	2.16E-06	Lipoyl-binding domain-containing protein
T265_03601	3.39	7.45E-03	G_PROTEIN_RECEP_F1_2 domain-containing protein
T265_12627	3.24	8.82E-04	PBPe domain-containing protein
T265_10342	3.17	5.91E-08	PDZ domain-containing protein
T265_10111	3.12	3.63E-12	SCP domain-containing protein
T265_10691	3.11	6.37E-05	G_PROTEIN_RECEP_F1_2 domain-containing protein
T265_15375	3.10	1.56E-52	Legumain^a^
T265_15407	3.08	5.38E-09	Legumain^a^
T265_12947	3.04	2.34E-06	guanylate cyclase
T265_15658	3.02	6.16E-06	PID domain-containing protein

^a^Genes involved in the proteolysis of the *O. viverrini* in the developing juvenile stage (D14).

**Fig 4:**
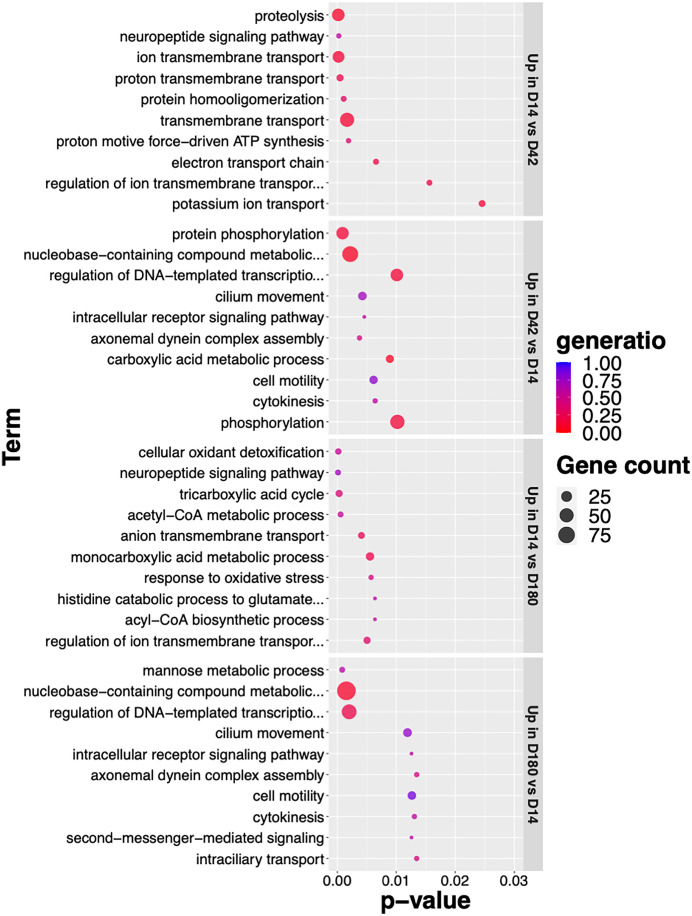
Top 10 enriched GO terms of up-regulated genes in the juvenile and adult stages. Only the biological process (BP) GO terms with p-value < 0.05 are shown, ranked by p-value. Dot size represents the number of genes associated with each BP term. Color represent gene ratio, i.e., the number of DEGs divided by the total number of genes annotated with the GO terms. The plot shows enriched biological processes of genes that were up-regulated from four pairwise comparisons: D14 *vs D42*, D42 *vs* D14, D14 *vs* D180, and D180 *vs* D14. Detailed results of all enriched terms and associated up-regulated genes are available in S4 Table.

Functional analysis using GO enrichment supported that the up-regulated genes of juvenile (D14) *O. viverrini* were enriched in the proteolysis process. With 37 up-regulated genes annotated with the GO term, examples of these are *metalloendopeptidase, peptidase M12B, peptidase family M13, Peptidase C13 family, trypsin, legumains, peptidase A, peptidase A1, peptidase* *S1**, peptidase_M14* (Fig 4 and S4 Table). These findings further corroborate the notion that a diverse peptidase gene, in addition to promoting energy utilization, may also contribute to their successful host invasion and immune modulation within hostile environment of the host’s bile duct [41–44]. In line with this, the neuropeptide signaling pathway was also enriched among the D14 up-regulated genes, including three genes related to G-protein coupled receptors (T265_00683, T265_00777, T265_06423) and a putative neuroendocrine protein with proteolytic function (T265_14272) (Fig 4 and S4 Table).

During the juvenile stage, several genes known to be related to development and cell differentiation, which require strict signaling regulation, were highly expressed. These include genes encoding *Homeobox (Hox) domain-containing protein* (T265_04509), *PI3K/PI4K domain-containing protein* (T265_14809), and *TGF-β domain-containing protein* (T265_13153 and T265_05750). Other genes representing various points in signaling cascades were also up-regulated in D14 including *guanylate cyclase* (T265_12947), *Protein tyrosine phosphatase* (T265_12471, T265_12698), *Ras family protein* (T265_10821, T265_15294), *Ras-like protein family member 10B* (T265_09665)*, Ras-like GTP-binding protein Rho1* (T265_09132), *Phosphoinositide interaction domain* (*PID) domain-containing protein* (T265_15658), and *cAMP-dependent protein kinase* (T265_09755) (S1 and S2 Tables). These findings underscore the critical role of diverse signaling pathways in early developmental processes and successful host adaptation of juvenile *O. viverrini*, likely facilitating coordinated regulation of growth, differentiation, and environmental sensing during establishment in the host.

Our results in D14 worms further revealed up-regulated genes involved in energy metabolism including *Pyruvate dehydrogenase E1 component subunit alpha* (T265_07717), *Pyruvate dehydrogenase E1 component subunit beta* (T265_11829), *Dihydrolipoamide acetyltransferase component of pyruvate dehydrogenase complex* (T265_00275), *Succinate-CoA ligase [ADP/GDP-forming] subunit alpha, mitochondrial* (T265_00432), *Cytochrome* (T265_09703), *Cytochrome c domain-containing protein* (T265_09199), *Cytochrome c oxidase subunit 1* (T265_12110), and *ATP synthase subunit beta* (T265_01732) (S1 and S2 Tables). These expression patterns suggest an increased demand for pyruvate, likely coming from host environment, tricarboxylic acid (TCA) cycle activity, and oxidative phosphorylation in D14, consistent with several enriched GO terms among the top 10, such as acetyl-CoA metabolic process, TCA cycle, electron transport chain, and proton motive force-driven ATP synthesis (Fig 4 and S4 Table). This highlights the energy-intensive nature of the early juvenile stage, reflecting a reliance on protein and carbohydrate metabolism for survival and maintenance.

The pairwise comparison between D42 and D180 adult worms showed only slight differences. Only five genes were considered significantly up-regulated in D42, and another five were up-regulated in D180 (S3 Table). The up-regulated genes at D42 included *H/ACA ribonucleoprotein complex subunit* (T265_11159), *Legumain* (T265_11946, T265_15375), *TGF_BETA_2 domain-containing protein* (T265_13153), and *Immunoglobulin domain protein* (T265_14313) (S3 Table). Four of these genes, excluding T265_11159, exhibited a declining expression pattern, with the highest expression observed at D14, followed by an approximately 2.5-fold decrease at each subsequent timepoint.

In contrast, the up-regulated genes at D180 in *O. viverrini*, associated with long-term infection included *L-ornithine N(5)-monooxygenase (NAD(P)H)* (T265_05877), *FHA domain-containing protein* (T265_06167), *Integrase catalytic domain-containing protein* (T265_10528), *Rho-GAP domain-containing protein* (T265_15550), and *Transcription elongation regulator 1* (T265_15863) (S3 Table). Again, all these genes demonstrated a steady increase in expression from D14 through D42 to D180, suggesting significant changes occurring in the parasites during chronic infection.

### Mating system and host immune evasion at advanced life stages

**Fig 5:**
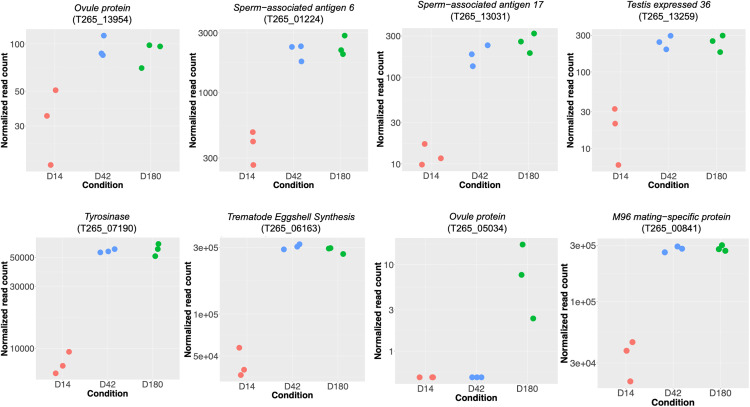
Gene expression of reproduction-related genes highly expressed in adult stages. Gene count plots show normalized read counts (DESeq2) of selected genes potentially involved in the reproductive system. Each dot represents a biological replicate.

Compared to the D14 juvenile worms, up-regulated genes in two adult stages (D42 and D180) showed, as expected, multiple genes potentially related to the parasite reproductive system. Given that this dataset came from pools of worms and their hermaphroditic nature, it is limited in describing the reproductive biology of the worms. However, their expression levels indicated that the gene expression patterns were consistently up-regulated in similar manners at both D42 and D180 compared to lower expression in D14 (Fig 5), emphasizing their likely roles in the reproductive stage of the parasite. These genes comprised of *Testis expressed 36* (T265_13259, T265_15840, T265_11057, T265_13259), *Meiosis-specific nuclear structural protein 1* (T265_12750), *Spermatogenesis associated 6 like* (T265_14145), *Sperm-associated antigen 6* (T265_01224), *Sperm-associated antigen 17* (T265_13031), *Ovule protein* (T265_13954, T265_03571, T265_10863), *Trematode Eggshell Synthesis* (T265_06163, T265_14710, T265_01554, T265_06806), *Tyrosinase* (T265_07190), *Tyrosinase_Cu-bd domain-containing protein* (T265_08937, T265_07191), and *M96 mating-specific protein* (T265_00841).

**Fig 6:**
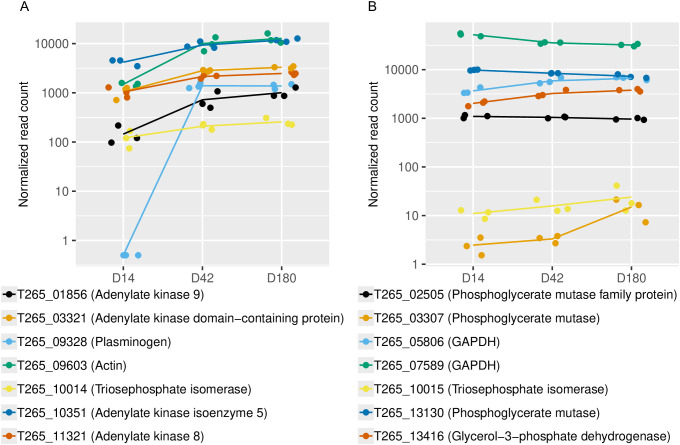
Expression of plasminogen and plasminogen-binding protein. Normalised counts of genes encoding plasminogen and plasminogen-binding proteins over the timecourse. Color represents different gene ID as shown under each panel. Gene descriptions are based on annotation from *O. viverrini* reference genome [17] on WBPS19. **A)** Genes that were up-regulated in at least one adult stage compared to D14 juvenile. **B)** Genes that were not considered up-regulated in adult stages compared to D14 juvenile. Details of log2FC and adjusted p-value for each pairwise comparison are available in S5 Table.

At D42 and D180, genes potentially related to host immune evasion were strongly up-regulated. In particular, a gene encoding *plasminogen* (T265_09328) showed a high expression level with log2FC greater than 12 (more than 4,000-fold increase) in both D42 and D180 conditions compared to the D14 group as clearly depicted in Fig 6A, light blue line. Plasminogen is a zymogen (proenzyme) of plasmin, which contributes to the mechanism of fibrin destruction. By the work of plasmin and its interacting proteins, blood clotting and extracellular matrix (ECM) can be destroyed, and this process has been related to the escape from the host immune system [45].

Moreover, genes with potential binding interactions to plasminogen [45–47] were up-regulated in both adult stages in our transcriptomic data compared to the D14 group. These genes include *Actin* (T265_09603), *Adenylate kinase 8* (T265_11321), *Adenylate kinase isoenzyme 5* (T265_10351), *Adenylate kinase domain-containing protein* (T265_03321), and *Adenylate kinase 9* (T265_01856) – all of which were up-regulated by 2–5-fold (Fig 6A and S5 Table). One of the *Triosephosphate isomerase* (T265_10014) also narrowly passed the cut-off for the pairwise comparison between D14 and D180 worms with log2FC close to 1-fold (S5 Table). Although the expression of other genes encoding plasminogen-binding proteins was not significantly different between juveniles and adults in our study, many of these genes only narrowly missed the cut-off for either log2FC or adjusted p-value. These genes include *Glyceraldehyde-3-phosphate dehydrogenase* (GAPDH; T265_07589, T265_05806), *Glycerol-3-phosphate dehydrogenase* (T265_13416), *Phosphoglycerate mutase* (T265_13130, T265_03307), *Phosphoglycerate mutase family protein* (T265_02505), and one of the *Triosephosphate isomerase* (T265_10015) (Fig 6B and S5 Table). Some of these enzymes are multifunction proteins. For example, GAPDH is an important enzyme in glycolysis, but it has been shown to be a key player in host-parasite interactions from bacteria, fungi, to parasitic worm [48,49] specifically by binding to host plasminogen and disrupt immune invasion and blood-clot lysis. This important multifunction nature may explain the non-concordance level with plasminogen, and spectacularly high expression level.

Among the up-regulated genes with dramatic differential regulation revealed a group of *SCP-like protein* (T265_14885) and *SCP domain-containing proteins* (T265_02482, T265_15023, T265_04266, and T265_12675), with log2FC > 10 or greater than 1,024-fold in adult stages compared to juvenile (S6 Table). The SCP-domain containing protein, also known as cysteine-rich secretory protein/antigen 5/pathogenesis related-1 (CAP) or Sperm-coating protein/Tpx-1/Ag5/PR-1/Sc7 (SCP/TAPS) domains has been recognized as a key virulence factor in parasitic infection [50]. The exceptionally high expression of these genes in adult stages from our study in both acute and chronic infection suggested that the *SCP-like protein and SCP domain-containing proteins* might be playing critical biological roles in the *O. viverrini* biology and survival.

**Fig 7:**
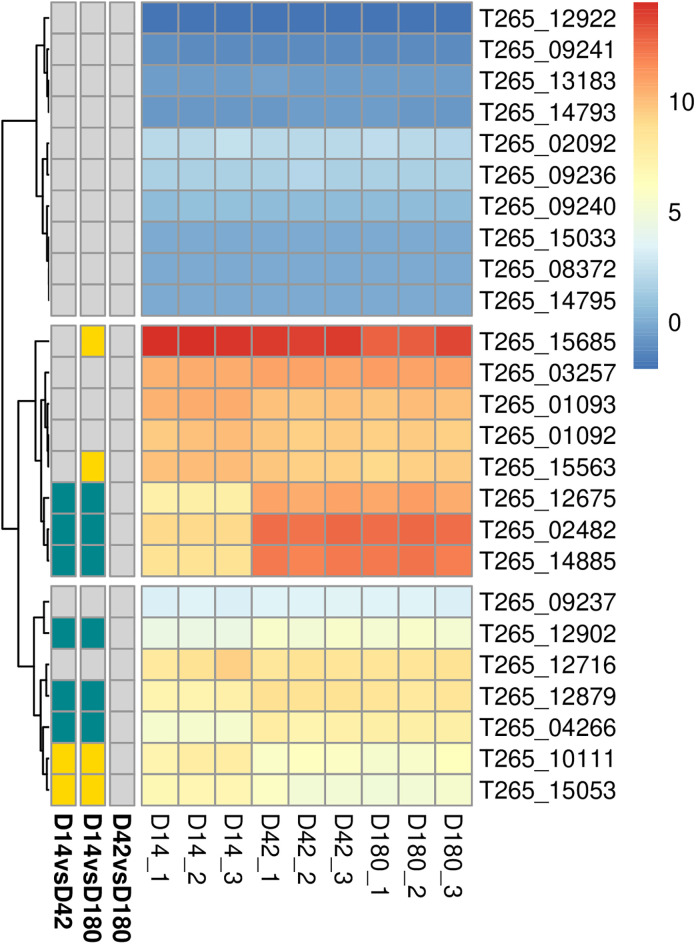
Hierarchical clustering of SCP/TAPS genes based on expression level over the timecourse. Heatmap color represent rlog-transformed values of SCP/TAPS genes that were assigned with protein family ID PF00188 (Pfam) and IPR014044 (InterPro). Annotation on the left indicated results of pairwise comparison (log2FC > 1 or <-1, and adjusted p-value < 0.01). Yellow indicates up-regulation in earlier timepoint (e.g., up-regulated in D14 compared to D42); turquoise indicates up-regulation in later timepoint; grey indicates not passing the cut-offs for differential expression. Details of log2FC and adjusted p-value for each pairwise comparison and based mean expression level are provided in S6 Table.

We further investigated the genes encoding SCP domain-containing proteins in *O. viverrini* by pulling expression profile of other genes in this family based on annotated protein domain (Pfam ID PF00188 and InterPro ID IPR014044) [50]. A total of 25 SCP/TAPS genes were found from reference genome of *O. viverrini* (Fig 7 and S6 Table). Note that there are other genes annotated as SCP domain containing protein or SCP-like protein; however, some of these genes were not assigned with the established protein domain ID associated with SCP/TAPS and therefore not included in our investigation. Of the 25 SCP/TAPS genes investigated, ten were express at very low level during the intra-mammalian stages in this study, rendering noisy data and insignificant adjusted p-value (Fig 7, uppermost cluster). Three of these showing no detectable reads at all. The rest seem to be expressed throughout the whole timecourse, but the level was shifting between timepoints. In particular T265_15685, show the highest expression throughout all three timepoints with the level dropped slightly at D180 (~ 3-fold compared with D14 and ~2 fold compared with D42). Most differentially expressed SCP/TAPS were up-regulated in the adult stages (both D42 and D180) (Fig 7, turquoise annotation), while some were highly expressed in D14 and reduced in expression down in adult worms (Fig 7, yellow annotation). This data emphasized SCP/TAPS genes that may be important for intramammalian infection, with some specific to juvenile or adults, and provide novel revenue for future investigation.

In advanced developmental stages (D42 and D180 compared to D14), up-regulation of other genes encoding products potentially related to immune interactions were observed. These include *Ig-like domain-containing protein* (T265_01038, T265_10657), *IL4_i_Ig domain-containing protein* (T265_01060), *Peptidase_M24 domain-containing protein* (T265_12477), and *Peptidase_M13_N domain-containing protein* (T265_12599, T265_15775) (S1 and S2 Tables). Some of these genes have orthologs in other parasitic fluke species, including *Opisthorchis felineus*, *Clonorchis sinensis*, and *Fasciola hepatica* (S7 Table), suggesting their potential roles in host-parasite interactions.

### Potential drug targets among cell surface receptors in juvenile and adult *O. viverrini*

At D14, almost 20 up-regulated genes were annotated as cell surface receptor annotated as *G_PROTEIN_RECEP_F1_2 domain-containing protein* (T265_03601, T265_10691, T265_02329, T265_01275, T265_03494, T265_02724, T265_00716, T265_00683, T265_09662, T265_14292, T265_08353, T265_11536, T265_01578, T265_06423, T265_01985, T265_00777, T265_02701) (S1 and S2 Tables). GO enrichment results support the inference that the majority of these GPCRs were involved in neuropeptide signaling pathway, and G protein-coupled peptide receptor activity (Fig 4 and S4 Table). In addition, the enriched GO term ion transmembrane transport contained 31 up-regulated genes in D14 worms, such as *ATPase protein 9*, *ATP synthase subunit beta*, *BTB domain-containing protein*, *Amiloride-sensitive sodium channel*, *Glycine receptor subunit beta-type 4*, *Calcium-transporting ATPase*, *Amino acid transporter*, *Neurotransmitter-gated ion-channel*, *Otopetrin*, and *Cytochrome c oxidase subunit 1* (S4 Table). GPCRs are known to be effective pharmacological targets in a various biological system. These upregulated GPCRs and ion channels may affect the parasite’s neuromuscular functions, sensory perceptions, or ability to absorb molecules from host, providing a rational basis for the development of antiparasitic medications.

In D42 and D180 worms, some *nuclear receptor domain-containing protein* (T265_09674, T265_15765) were revealed in enriched GO term related to transcriptional regulation and DNA-binding and nucleobase-containing compound metabolism (Fig 4 and S4 Table), suggesting roles in stage-specific gene expression regulation. In parallel, multiple genes related to protein phosphorylation and signaling pathway regulation were also up-regulated in D42 and D180, including *receptor protein-tyrosine kinases* (T265_03081 and T265_02140) in D14, and *protein-tyrosine phosphatases* (T265_10810, T265_10484, T265_01620, T265_13563, T265_13236) (S1 and S2 Tables). Consistently, the top enriched GO terms at D42 and D180 included protein phosphorylation, representing many members of *protein kinase domain-containing proteins* (Fig 4 and S4 Table). These findings point to dynamic regulatory processes during parasite maturation and chronic infection, and further highlight candidate genes, particularly kinases, phosphatases, and nuclear receptors, which have been established as drug targets in oncology and immunomodulation, where a range of small-molecule inhibitors is already available.

### GSEs across the life stages of *O. viverrini* in the hamster model

**Fig 8:**
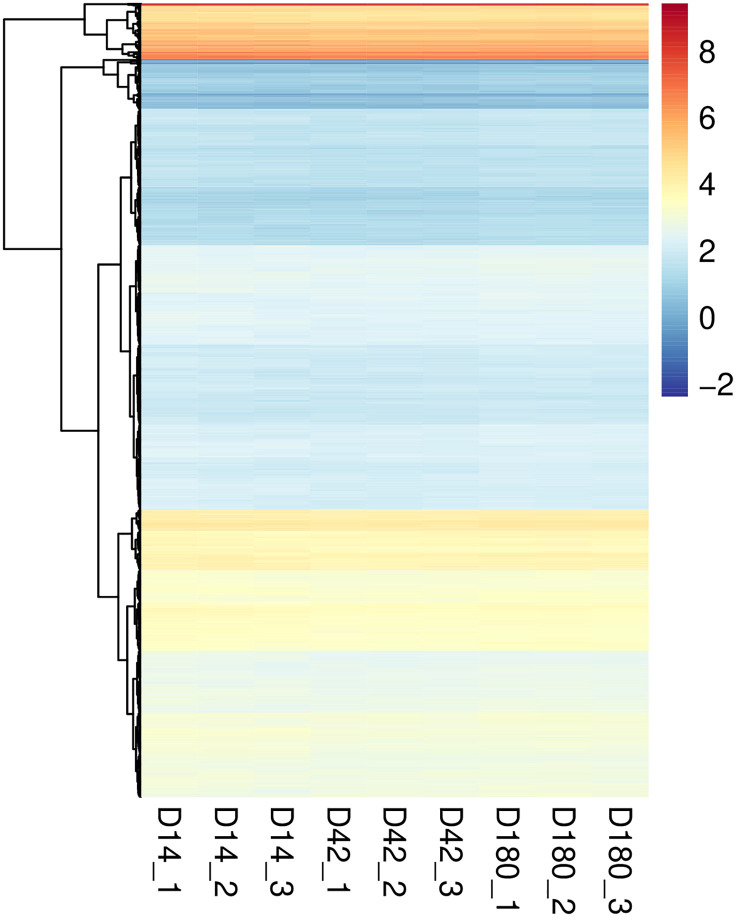
Novel 2,011 GSEs in *O. viverrini* during intramammalian infection. GSEs are those with CV ≤ 0.15 across all nine samples (three timepoints, three replicate each). Color of the heatmap represent log TPM. The X-axis showed different sample groups, the Y-axis represent the identified GSEs. Full details with gene IDs, gene description, TPM, and CV values are provided in S8 Table.

While differentially expressed genes highlight stage-specific changes and putative virulence factors in *O. viverrini*, identifying genes consistently expressed across intramammalian stages may reveal novel diagnostic biomarkers and further identify drug targets. To investigate GSEs, raw gene count values were converted to TPM, and CV were calculated for each gene across all replicates of all timepoints. As a result, a total of 2,011 *O. viverrini* genes passed the criteria for GSEs (CV ≤ 0.15), indicating that these GSEs were constitutively expressed across all nine samples. However, as demonstrated on the heatmap, the level of expression varied with a few genes expressed at exceptionally high levels and the majority showing relatively low expression (Fig 8).

**Fig 9:**
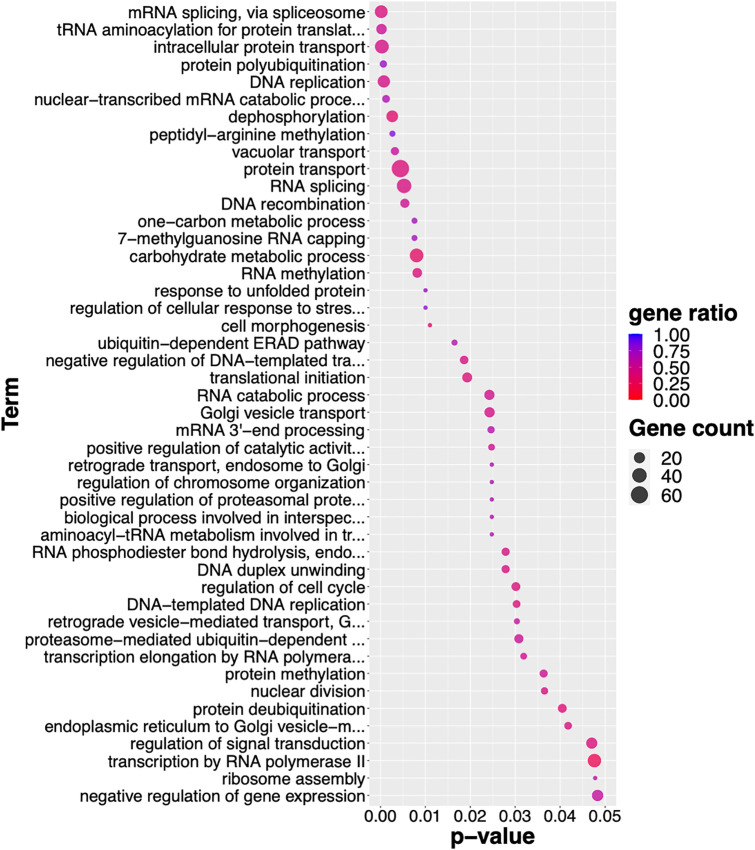
Enriched GO terms of GSEs. Only biological process terms with p-value < 0.05 are shown, ranked by p-value. Dot size indicates the number of genes associated with each GO term. Color represents gene ratio which is the number of GSEs divided by the total number of genes annotated with each GO term. Detailed results of all enriched terms and associated GSEs are available in S9 Table.

Notably, we conducted the GO enrichment analysis to comprehend all fundamental cellular processes derived by GSEs that impact to the parasite biology. The GO enrichment analysis of GSEs revealed around total hundred terms related to biological processes (BP), molecular functions (MF), and cellular component (CC) (S9 Table). In terms of BP, the GSEs were associated with the fundamental flow of genetic information from DNA to RNA to protein such as DNA replication and mRNA processing. Some of the top-scoring enriched BP were related to RNA metabolisms and modification. In addition, the transport of cellular materials and biological molecules was evidently noted in BP, such as protein transport, Golgi vesicle transport, exocytosis. Additionally, the regulation of cell cycle and cellular response were also observed (Fig 9 and S9 Table). Our data suggest that all these biological processes are crucial for *O. viverrini* during their intra-mammalian development.

**Fig 10:**
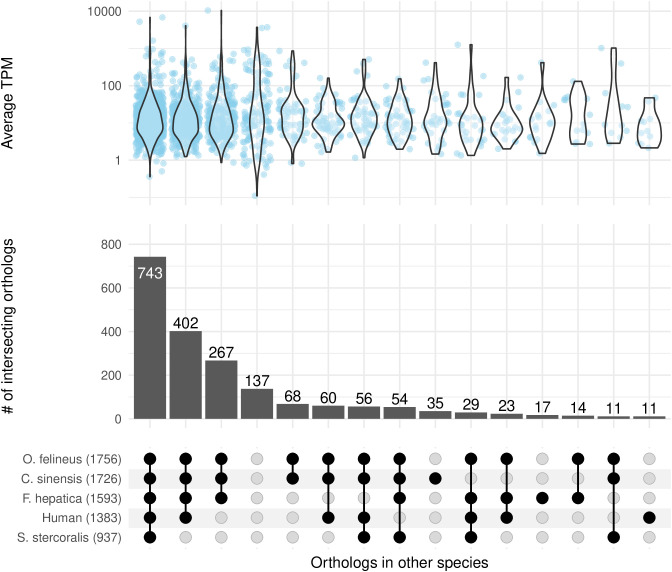
Shared orthology in GSEs. The bottom upset plot shows presence of GSEs in *O. viverrini* in other relevant species – human, *O. felineus*, *C. sinensis*, *F. hepatica,* and *S. stercoralis* – as indicated by black dots. The number after each species names indicates the total number of orthologs found among the 2,011 GSEs in *O. viverrini*. The correspondent bar chart indicates the number of intersecting orthologs across species. The upper violin plot represents distribution of GSE expression level in our *O. viverrini* data shown as average TPM from all replicates of all timepoints. The identification of inter-species orthology was based on available public data provided by WBPS19 [33]. Details of gene IDs and gene descriptions in *O. viverrini* and correspondent orthologs are available in S8 Table.

Of a total 2011 GSE, those with potential value as biomarkers for diagnosis or novel candidate for drug targets were identified based on existence of gene orthologs in other relevant species including human host, and closely related trematode *O. felineus*, *C. sinensis*, *F. hepatica*, and common co-infection agent *Strongyloides stercoralis* (Fig 10 and S8 Table). The highest number of GSE orthologs were found from *O. felineus*, followed by *C. sinensis*, both of which are closely related trematodes, and followed by *F. hepatica*, another liver fluke. This pattern is consistent with phylogenetic relationships. Interestingly, more GSE orthologs were shared with humans than with *S. stercoralis*, a parasitic nematode. A likely explanation is that *S. stercoralis* has a small genome (~43 Mb), compared to trematode such as *O. viverrini*, *O. felineus*, *C. sinensis*, and *F. hepatica* (~500–2000 Mb) and has undergone substantial gene loss and divergence, especially in conserved pathways, as part of its specialized parasitic lifestyle. As a result, its compact genome and annotation limitations may reduce detectable orthologs. Comparative genomics of major parasitic worms have demonstrated that variations in genome size influence gene contents, resulting in many gene families being highly diverged where ancestry is difficult to traced [51]. Furthermore, evolutionary analysis demonstrated that gene loss is less captured in Lochotrophozoa (which includes platyhelminthes) than Ecdyzozoa (which includes nematodes) [52]. This low GSE conservation is also reflected by the evolutionary distance separating Lophotrochozoa and Ecdysozoa.

We identified 137 GSEs with no detectable orthologs in any of the species examined (Fig 10 and S8 and S10 Tables). *S. stercoralis* was included due to its common co-endemicity with *O. viverrini* in rural areas, where populations are frequently exposed to soil and natural water sources. These 137 uniquely expressed genes could serve as specific targets for *O. viverrini* detection by protein antigen-based diagnostics – an approach already validated and approved by the Thai FDA and used in field settings [11]. These genes warrant further investigation, including proteomic profiling or validation using archived or prospective samples from infected individuals. On the other hand, for drug target development, GSEs conserved across multiple fluke species may be more valuable, as three trematodes (*O. viverrini*, *O. felineus*, and *C. sinensis*) are currently treated with praziquantel, with risk of reduced efficacy in regions with heavy use. To this end, we highlighted 267 constitutively expressed GSEs that are conserved in other liver fluke species as potential multi-species drug targets (Fig 10 and S8 and S11 Tables).

**Fig 11:**
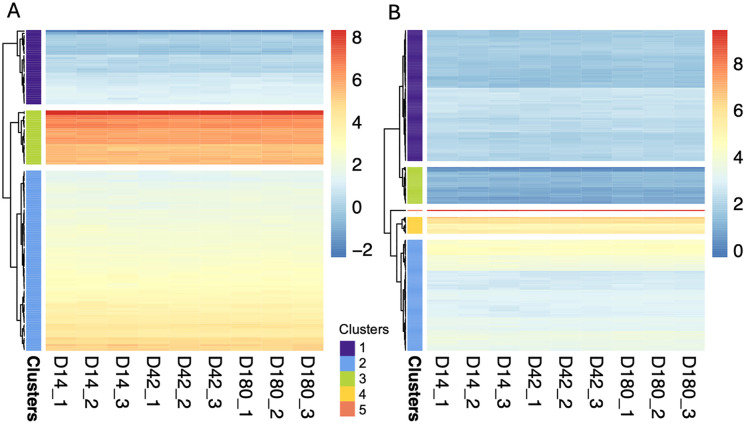
Clusters by expression level of GSEs. Color of the heatmaps represents log-transformed TPM. Cluster numbers were assigned internally during hierarchical clustering. **A)** Expression level of GSEs with no detectable orthologs in any of the species examined. **B)** Expression level of GSEs conserved in other liver fluke species. Details on the expression levels of each GSE (average TPM) and the grouping cluster they belong to are provided in S10 and S11 Tables.

Expression of these genes could be useful information inferring the suitability as a diagnostic biomarker. We, therefore, clustered the genes found in the *O. viverrini*-only group (137 genes) and identified some groups of highly expressed genes (Fig 11A and S10 Table); for example, *RRM domain-containing protein* (T265_16372 (645.87 TPM)), *eIF3g domain-containing protein* (T265_16333 (723.07 TPM)), *LIM domain protein* (T265_14316 (384.14 TPM)), and *Fibronectin type-III domain-containing protein* (T265_00431 (22.01 TPM)). There were, however, genes with apparently high TPM but without annotated description. Some of these genes, T265_13159 (3528.37 TPM) and T265_15973 (3695.81 TPM) for examples, are relatively short and maybe misannotated. Others without annotated description, however, could be of interest; for example, T265_12203 (229.47 TPM) and T265_04304 (457.56 TPM) consist of transmembrane domain and may be exposed to host environment and thus could serve as good candidates for diagnostic biomarkers. Genes with signal peptide suggested that they may be released into host environment, such as T265_10887 (19.21 TPM) and T265_11396 (annotated as *lysosome-associated membrane glycoprotein 2*, 7.04 TPM) and may serve as diagnostic biomarkers.

Likewise, genes with potential value as novel drug targets – shared among related liver flukes – also show varying expression level (Fig 11B and S11 Table). Of the 267 genes, identified as having shared orthologs with other liver fluke species but not with humans, several of these genes are involved in critical cellular processes such as RNA transcription, protein stability, and enzymatic activity, which are crucial for the survival and pathogenicity of parasitic flukes. Notably, some of these genes encode enzymes that have known repurposable drugs, suggesting their relevance for therapeutic targeting; for instance, *cytochrome P450* (T265_00657) and *fatty acid synthase* (T265_01246) [53]. Additionally, the clusters of expression level of these genes (S11 Table) offer insights into their functional roles and relevance for drug development. Targeting these conserved genes could pave the way for broad-spectrum treatments against parasitic flukes, improving therapeutic options across multiple species.

## Discussion

In this study, we presented a comprehensive transcriptome analysis of *O. viverrini* to identify differentially expressed genes associated with the parasite developmental milestones within mammalian host. Notably, the up-regulation of genes involved in energy metabolism, and signaling pathways inferred mechanisms supporting the parasite’s survival and growth within the liver of hamsters. In adult stage, we revealed key up-regulated genes that might be influenced the mating and reproduction, suggesting the molecular basis for the parasite’s reproductive success in a monoecious organism. Additionally, our analyses revealed the expression of genes involved in host defense mechanisms, providing insights into the parasite’s strategies for evading the host immune response. For the first time, the GSEs of Asian liver fluke were explored in our study using RNA-seq analysis that include parasites from a chronic infection. These GSEs represented potential candidate genes for diagnostic applications and development of novel intervention against *O. viverrini* and related liver fluke infection. The challenge of our transcriptomic profiling was that some intermediate mRNAs might not be produced as functional proteins depending on many factors, such as mRNA isoforms, alternative splicing, post-translation modification. As a result, the technologies of protein-based identification could be further implemented using additional *in vivo* specimen.

During the development of juvenile (D14), diverse peptidase genes related to proteolysis were considered to promote the evasion of host immune system and parasite feeding [54]. However, it could also be related to developmental period of the parasites as multiple genes related to signalling and differentiation were also up-regulated. *Homeobox* genes, or well-known as *Hox*, are associated with growth and development by acting as transcription factors [55] in model organism as well as in parasitic worm, such as *Drosophila melanogaster* (fruit fly) and *Schistosoma mansoni* (human blood fluke) [56]. High expression of *PI3K domain-containing protein* has been described previously in *F. hepatica* at immature liver stage, responding for rapid differentiation and proliferation of stem cells [57]. Up-regulated *guanylate cyclase*, *cAMP-dependent protein kinase*, and *TGF-β domain-containing protein* in juvenile worms from our dataset infer a wide range of cellular functions, including signal transduction in cell growth and development via second messenger molecules, cAMP and cGMP [58]. For instance, in *C. elegans*, cGMP and TGF-β signaling play roles in regulation of behavior of food intake [59].

Multiple signaling-related genes were up-regulated in juvenile *O. viverrini*, and these have been proposed as drug targets as listed in the study of *S. mansoni* genome and *Taenia pisiformis* transcriptome, such as GPCRs, voltage-gated ion channels, kinases, and neuropeptides [38,60]. Specifically, inhibitory small molecules against certain proteases tends to be developed as potential vaccine targets for the prevention of helminth infection [61]. Our RNA-seq data revealed many up-regulated genes in each developmental stages with roles in cell signaling. These candidate genes could contribute as drug or vaccine targets, resulting in the interruption of many internal signal transduction pathways as well as terminating the important biological functions in *O. viverrini*.

At more advanced life stage (D42 and D180), up-regulation of multiple reproductive genes is consistent with the mature stage of the parasite. Transcriptomic profiling of the *S. mansoni* found the eggshell production-related genes which were *tyrosinase* and *egg shell protein* being up-regulated in adult human blood fluke [39]. Another work using the RNA-seq data of *S. mansoni,* revealed that *tyrosinase* was proposed as a component of eggshell synthesis [62]. Surprisingly, a gene encoding *ovule protein* showed low expression level in the juvenile stage despite the worms supposedly being reproductively inactive. This might be a novel information of *O. viverrini* relating to its egg production prior to fully mature adult stage, or the gene may have multiple functions. Moreover, this information may offer an alternative strategy for intervention aimed at parasite burden control by focusing on reproductive biology as demonstrated in species like *S. mansoni* [63]*.*

Multiple genes encoding SCP domain-containing proteins showed extremely high expression levels at D42 and D180 compared to the juveniles, and suggested candidate SCP/TAPS proteins that maybe important for chronic infection of *O. viverrini*. According to related studies, diverse SCP/TAPS proteins in parasitic helminth species were identified. For example, *venom allergen homologue asp-like 1* (GenBank accession number: AF334661) in *Brugia malayi* contains a single SCP-domain and is involved in mammalian host invasion process [50,64]. Additionally, in *Ancylostoma caninum*, two secreted proteins with SCP/TAPS domain (secreted protein-2 (GenBank accession number: Q16937) and secreted protein-3 (GenBank accession number: AY217004) are involved in pathogen recognition during infection process and maintenance of host-parasite interaction [64]. Notably, the proteomic analysis in hookworm, *Necator americanu*s revealed proteins sharing SCP domain-containing proteins, such as NAME_01772 (asp1 (SCP-like protein)), NAME_01050 (SCP domain-containing protein), and NAME_06207 (CAP superfamily) that might be associated with functional roles of SCP/TAPS family [65].

Significantly and strongly up-regulated in both adult stages compared to the juvenile stage included a gene encoding *plasminogen* (T265_09328) and some putatively plasminogen-binding proteins. Plasminogen has been studied for their crucial roles in host immune response in other species of trematode, particularly for its fibrinolytic and host immune evasion activity. In *Schistosoma bovis*, tegument extracts of mature male worm were able to increase plasmin production similar to tissue-type plasminogen activator. Their proteomic results revealed ten plasminogen-binding proteins [47]. During the adult stage of sheep liver fluke (*F. hepatica*), the expression of *plasminogen-binding protein* degraded blood clotting via action of bradykinin, resulting in host inflammation and chronic fascioliasis [46]. What is more, the investigation of *serine protease 2* in *S. mansoni* secretome suggest the tendency of converting plasminogen to plasmin by the parasite, which in turn affect thrombolysis, and modulate host immune defense via anti-hemostatic properties [66].

Principle component analysis revealed that the two adult stages (D42 *vs* D180) differ by only 2% of the variance, indicating minimal transcriptomic changes during chronic infection. Consistent with this, only 10 genes were differentially expressed between the two stages, suggesting that adult *O. viverrini* maintain largely stable gene expression profiles once maturity is reached. Minor expression changes, such as up-regulation genes related to RNA processing (*H/ACA ribonucleoprotein complex subunit*) and immunomodulation (*legumain*, *TGF-ß2-domain containing protein,* and *immunoglobulin domain protein*) may reflect subtle host-parasite interactions rather than major developmental transitions. These findings support the notion that factors beyond the parasite’s life cycle development, such as host immune state or tissue microenvironment, may contribute more substantially to chronic infection outcomes [67].

To identify candidate genes for liver fluke intervention, we identified over 2,000 GSEs that were constitutively expressed during mammalian infection. Of these, the most abundance proteins comprised genes encoding *tubulin*, *heat shock protein 90* (*HSP90*), and *fructose-bisphosphate aldolase*. These proteins was suggested as vaccine targets with high antigenic activity in fish ectoparasitic flatworm (monogeneans) [68]. *Tubulin-specific chaperone D* in *O. viverrini* is highly similar to that of *F. hepatica*, *C. sinensis*, and *O. felineus*, but with low percent identity to human [33]. This gene also have the constant expression level in all developmental phases of *F. hepatica* [69], suggesting its value as potential new drug or diagnostic candidate against liver flukes. Moreover, our identified GSEs could be employed as reference gene in quantitative assays such as RT-qPCR. A gene *cAMP-dependent protein kinase* was suggested as reference gene of tapeworm, *Hymenolepis microstoma*, for the gene quantification by RT-PCR. It was found to be more suitable for comparison with low-expression genes, such as *Hox*, because its Ct values is relatively close to that of *Hox*, resulting in less potential confounding effect when using high-expression genes such as *18S rDNA* as a reference control [70]. It worth emphasizing that our list of GSEs provide genes covering a range of expression level, which can be utilized appropriately as a reference for genes of interest.

Our study provides novel insights into the biology of *O. viverrini*, spanning from the juvenile stage to the acute- and chronic-infected adult stages within mammalian hosts. Transcriptomic analysis revealed numerous stage-specific genes that are critical for the survival and development of *O. viverrini*, highlighting potential therapeutic targets for disrupting key biological processes and advancing diagnostic applications. Furthermore, the identification of genes with previously unknown functions underscores the informed opportunity for future investigations to explore their roles in parasite biology. Overall, this work enhances our understanding of the molecular landscape of *O. viverrini* and establishes a valuable foundation for the development of innovative diagnostic and preventative strategies against opisthorchiasis.

## Conclusion

In conclusion, our RNA-seq analysis of intra-mammalian stages *O. viverrini* has significantly expanded our understanding of the parasite’s biology across its developmental stages. By investigating the gene expression profile, we have identified key up-regulated genes in biological processes, including energy metabolism, proteolysis, signaling pathways, and growth and development. Furthermore, our findings have revealed numerous genes associated with the parasite’s survival within the mammalian host, including those involved in evading and interacting with the host immune response. Notably, we identified 2,011 genes with stable expression, essential for maintaining cellular functions, some of which are uniquely present in non-human species. The transcriptomic data from this study not only provide a deeper understanding of *O. viverrini* infection, but also offer a valuable resource to support future research and development of anthelmintic targets and diagnostic markers for *O. viverrini* and other related parasitic worms.

## Supporting information

S1 Table Pairwise comparison of differentially expressed genes between D14 and D42. (XLSX)

S2 Table Pairwise comparison of differentially expressed genes between D14 and D180. (XLSX)

S3 Table Pairwise comparison of differentially expressed genes between D42 and D180. (XLSX)

S4 Table GO enrichment results of up-regulated genes in each condition. (XLSX)

S5 Table Log2FC and adjusted p-value of plasminogen and genes previously proposed or characterized as plasminogen-binding protein in *O. viverrini* or related species. (XLSX)

S6 Table Log2FC and adjusted p-value of SCP/TAPS genes (PF00188 (Pfam) and IPR014044 (InterPro)). (XLSX)

S7 Table Orthologues of *O. viverrini* immune-related genes in *O. felineus*, *C. sinensis*, and *F. hepatica.* (XLSX)

S8 Table TPM, CV, and orthology information of 2,011 GSEs in *O. viverrini* during infection in the mammalian host. (XLSX)

S9 Table GO enrichment results of GSEs. (XLSX)

S10 Table Expression level and cluster grouping of GSEs with no detectable ortholog in any of the species examined. (XLSX)

S11 Table Expression level and cluster grouping of GSEs conserved in other liver fluke species. (XLSX)

S1 File Coding sequence counts for DEG analysis. The HTSeq-count data in the zipped folder contains the following files: ov_reads_D14_1_cds.txt, ov_reads_D14_2_cds.txt, ov_reads_D14_3_cds.txt, ov_reads_D42_1_cds.txt, ov_reads_D42_2_cds.txt, ov_reads_D42_3_cds.txt, ov_reads_D180_1_cds.txt, ov_reads_D180_2_cds.txt, ov_reads_D180_3_cds.txt.
(ZIP)
